# Polymorphisms and genetic susceptibility of type 1 diabetes mellitus and celiac disease

**DOI:** 10.1186/1758-5996-7-S1-A216

**Published:** 2015-11-11

**Authors:** Marília Dornelles Bastos, Thayne Woycinck Kowalski, Luiza Monteavaro Mariath, Marcia Khaled Puñales, Balduíno Tscheidel, Bibiane Armiliato de Godoy, Lara Dias Coutinho, Rafaela Fernandes Mundstock, Lavínia Schüler Faccini, Themis Reverbel da Silveira

**Affiliations:** 1 Universidade de Santa Cruz do Sul-UNISC, Porto Alegre, Brazil

## Background

Type 1 diabetes mellitus (T1D) is a chronic autoimmune disease characterized by pancreatic beta-cell destruction, hyperglycemia and progressive insulin deficiency, affecting mainly children and adolescents genetically predisposed. About 10% (2.4-16.4%) of T1D individuals develop celiac disease (CD), an immune-mediated enteropathy triggered by gluten exposure. Both diseases have a common autoimmune origin and share a similar genetic Background, the major histocompatibility complex class II antigen (HLA-DQ). Some genetic association studies have also identified susceptibility polymorphisms non-HLA associated to both diseases, located in different genes: RGS1 (rs2816316), IL2-IL21 (rs6822844), BACH2 (rs11755527) and IL18RAP (rs917997).

## Objective

The aim of the present study was to determine the allelic and genotypic frequencies of polymorphisms in RGS1, IL2-IL21, BACH2 and IL18RAP genes in a sample of 317 T1D individuals and to compare the frequency of the risk alleles in patients with negative (N=264) or positive (N=53) serology for CD.

## Materials and methods

Saliva or blood sample was collected and DNA extraction and genotyping performed by PCR Real-Time. All polymorphisms were in Hardy-Weinberg equilibrium. The comparison between the allele and genotype frequencies was calculate by Chi-square and Fisher's exact test.

## Results

The frequency of the allele risk for the genes RGS1 (allele A); IL2-IL21 (allele C); BACH2 (allele C) e IL18RAP (allele T) in T1D individuals and negative serology for CD was respectively: 95.5%, 97.4%, 79.8% and 46.6%. In T1D with seropositive, the frequency of the same alleles were: 98.1%(p=0.703), 100.0% (p=0.604), 86.0% (p=0.433) and 46.2%(p=1.000). Both genotype and allele frequencies were not significantly between T1D with negative or positive serology for CD.

## Conclusion

Our data did not evidence differences between the polymorphisms non-HLA analyzed in T1D with or without seropositive for CD, being not possible to assure that the presence of these polymorphism increase or decrease the predisposition to celiac disease.

